# Should We Abandon Intraperitoneal Chemotherapy in the Treatment of Advanced Ovarian Cancer? A Meta-Analysis

**DOI:** 10.3390/jpm13121636

**Published:** 2023-11-23

**Authors:** Maria Teresa Climent, Anna Serra, Carolina Balaguer, Antoni Llueca

**Affiliations:** 1 Multidisciplinary Unit of Abdominopelvic Oncological Surgery (MUAPOS), Department of Obstetrics and Gynaecology, Hospital General Universitario de Castellón, 12004 Castellón, Spain; serraa@uji.es (A.S.); antonillueca@gmail.com (A.L.); 2 Oncological Surgery Research Group (OSRG), Department of Medicine, University Jaume I (UJI), 12004 Castellón, Spain; 3 Department of Medicine, University Jaume I (UJI), 12004 Castellón, Spain; al3788436@uji.es

**Keywords:** advanced ovarian cancer, intraperitoneal chemotherapy, overall survival, disease-free survival, primary cytorreductive surgery

## Abstract

Background: Ovarian cancer is the gynaecological malignancy with the highest mortality and diagnosis often occurs in its advanced stages. Standard treatment in these cases is based on complete cytoreductive surgery with adjuvant intravenous chemotherapy. Other types of treatment are being evaluated to improve the prognosis of these patients, including intraperitoneal chemotherapy and antiangiogenic therapy. These may improve survival or time to relapse in addition to intravenous chemotherapy. Objective: The aim of this meta-analysis is to determine whether treatment with intravenous chemotherapy remains the gold standard, or whether the addition of intraperitoneal chemotherapy has a benefit in overall survival (OS) and disease-free interval (DFS). Materials and methods: A literature search was carried out in Pubmed and Cochrane, selecting clinical studies and systematic reviews published in the last 10 years. Statistical analysis was performed using the hazard ratio measure in the RevMan tool. Results: Intraperitoneal chemotherapy shows a benefit in OS and DFS compared with standard intravenous chemotherapy. The significant differences in OS (HR: 0.81 CI 95% 0.74–0.88) and in DFS (HR: 0.81 CI 95% 0.75–0.87) are statistically significant (*p* < 0.00001). There were no clinical differences in toxicity and side-effects. Conclusion: Intraperitoneal chemotherapy is an option that improves OS and DFS without significant toxicity regarding the use of intravenous chemotherapy alone. However, prospective studies are needed to determine the optimal dose and treatment regimen that will maintain the benefits while minimising side effects and toxicity and the profile of patients who will benefit most from this treatment.

## 1. Introduction

Ovarian cancer is one of the most frequent gynaecological tumours, ranking third. Ovarian cancer has a five-year survival of less than 20% [1,2]. Diagnosis is often delayed due to non-specific or absent symptoms, with most patients (70–80%) being diagnosed in the advanced stages of the disease [1,2,3]. The principal histological type is serous with a frequency of 75–80% [3].

The treatment of choice for advanced ovarian cancer (AOC) is cytoreductive surgery with the aim of achieving the absence of tumour residue (R0) followed by adjuvant intravenous chemotherapy treatment based on the combination of carboplatin and paclitaxel [4,5].

Due to delays in the diagnosis of ovarian cancer and the high probability of relapse in these patients, only 10–15% have a large disease-free interval [6]. It is necessary to find the best treatment to minimise the possibility of relapse and to increase overall survival.

Surgery aiming for the absence of tumour residue remains the primary objective in the management of advanced ovarian cancer. The definition of optimal cytoreductive surgery has evolved over the years. Currently, the absence of macroscopic tumoral residue is the objective in the primary cytoreductive surgery [4,7,8,9].

Chemotherapy treatment has been a controversial topic. The debate revolves around whether to administer the intravenous regimen (IV) alone (standard regimen) or with additional therapies such as intraperitoneal chemotherapy (IP) or other medications such as bevacizumab and PARP inhibitors (iPARP) with the aim of increasing survival and delaying the relapse [5,10,11].

The mechanism of action and the theoretical benefit of intraperitoneal chemotherapy lies in increasing the effect of the drugs in the peritoneal cavity and decreasing systemic toxicity [12,13].

There is a high degree of controversy surrounding the intraperitoneal administration of the chemotherapy agents, as well as a lack of consensus among different clinical guidelines [14,15,16].

Three randomised studies, (GOG 104 [17], GOG 114 [12], and GOG 172 [18]), published in 2001 and 2006, respectively, show statistically significant benefits in overall survival (OS) and disease-free interval (DFS) in intraperitoneal chemotherapy groups. The use of intraperitoneal chemotherapy has not been widespread, despite it showing the greatest increase in overall survival in ovarian cancer, reaching 16 months. Arguments for not using this treatment are based on concerns regarding toxicity symptoms, including asthenia, neurotoxicity, and abdominal pain. There are also difficulties associated with its administration and the use of the intraperitoneal chemotherapy catheter [12,19].

Currently, there is no quality evidence to recommend the use of intraperitoneal chemotherapy in the primary treatment of advanced ovarian cancer. It could be reserved for patients with FIGO stage III with complete or optimal cytoreductive surgery (absence of tumour or residual tumour less than 1 cm, respectively), with good performance status or in younger patients [20,21,22,23,24].

That is why the numbers of long-term overall survivors have been stable over the last 20 to 30 years, and there has not been an increase since platinum-based chemotherapy without a significant effect of new target treatments or increased surgical radicality [22].

There is a lack of consensus and high-grade evidence regarding the optimal treatment of advanced ovarian cancer.

The aim of this meta-analysis was to evaluate whether isolated intravenous chemotherapy still is the most efficacious regimen in patients with advanced ovarian cancer undergoing complete primary surgery.

## 2. Material and Methods

### 2.1. Search Strategy

This meta-analysis was carried out based on the indications drawn from the reporting items for systematic reviews and meta-analysis (PRISMA) guidelines (PRISMA 2020, http://www.primastatement.org, accessed on 1 September 2023). The Prisma Checklist is available in Supplementary Materials [23].

Pubmed and Cochrane central register of databases were searched for literature published between 2016 and February 2023 comparing the use of intraperitoneal chemotherapy added to intravenous chemotherapy in advanced ovarian cancer with the use of intravenous chemotherapy alone.

The search terms used were (‘Advance ovarian cancer’ AND ‘intravenous chemotherapy’ AND ‘intraperitoneal chemotherapy’). Controlled trials and systematic reviews were included. The hazard ratio of the intervention measure and the 95% confidence interval (CI) for OS and DFS were obtained from each study that met the inclusion criteria.

### 2.2. Inclusion and Exclusion Criteria

Inclusion criteria followed the PICOS criteria (population, intervention, comparison, outcome, and study design). The population studied was patients with advanced ovarian cancer undergoing primary cytoreductive surgery. We compared the adjuvant treatment administered: intravenous chemotherapy versus intravenous chemotherapy plus intraperitoneal chemotherapy and evaluated the outcomes DFS and OS.

Articles published in the last 10 years in English or Spanish were included. We excluded studies that lacked the rate of complete cytoreduction or applied hyperthermia; included the use of other drugs; or included patients with extra-abdominal disease.

### 2.3. Selection Process

The selection of articles was based on the critical reading of two review authors. Disagreements were resolved by discussion or with the help of a third review author.

The search was performed using the following filters:

- Languages: English and Spanish.
- Date: published articles since 2013.
- Type of study: controlled trial, systematic review, and meta-analysis.
- Exclusion of the articles that use hyperthermic intraperitoneal chemotherapy, neoadjuvant chemotherapy, other drugs, patients in the initial stages of ovarian cancer, and patients with extra-abdominal disease.

### 2.4. Statistical Analysis

The aim of the present study was to analyse whether intraperitoneal chemotherapy in patients with advanced ovarian cancer treated by cytorreductive surgery has a benefit in OS and DFS. The hazard ratio was used as a measure using a fixed-effect model. The results obtained are represented by forest plots graphs.

*p*-values of less than 0.05 were considered statistically significant.

Heterogeneity was assessed using the I2 tool. A value of less than 25% was considered low, 50% intermediate, and greater than or equal to 75% was considered high heterogeneity.

RevMan software was the tool used to evaluate the results (Review Manager (RevMan) Version 5.4, the Cochrane Collaboration, 2020).

## 3. Results

### 3.1. Selected Studies

**Figure 1:**
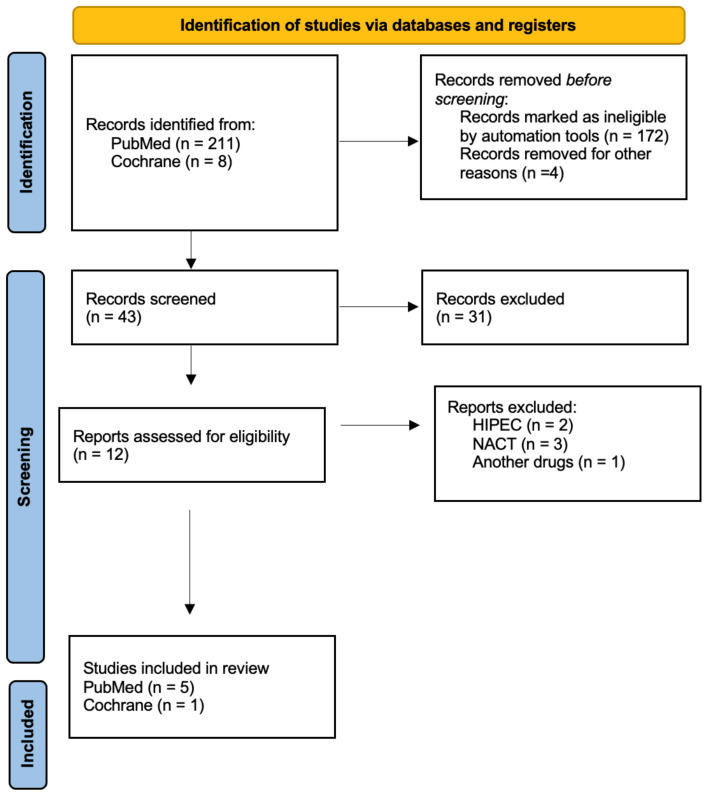
PRISMA diagram showing the selection process of the articles included in the study.

The search strategy obtained a total of 211 publications in PubMed and 8 in Cochrane. After the removal of duplicates, the filtering of articles with electronic tools, and the critical reading of the abstracts, five articles in PubMed and one article in Cochrane were selected. Six articles were included in this review for overall survival and five articles for disease-free survival. Figure 1 shows the selection process.

The six studies selected had a study population of 4465 patients for the overall survival analysis and 3348 for the disease-free survival analysis.

Table 1 and Table 2 show the main features of the study designs and the clinicopathological characteristics of the included population. Bias analyses are detailed in Figures S2 and S3 in the Supplementary Materials.

### 3.2. Results of Meta-Analysis on Overall Survival and Disease-Free Interval

Six articles included data on overall survival rates, but Wright et al.’s study did not evaluate disease-free survival rates.

**Figure 2:**
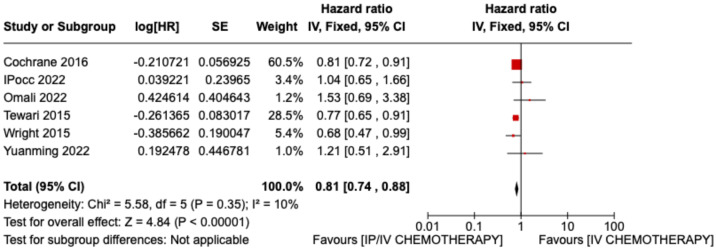
Statistical study of OS: Results obtained from all studies included in the review [13,24,25,26,27,28].

The analysis of the included data from each article shows an increase in overall survival in the group of patients treated with intraperitoneal chemotherapy compared with patients treated with standard intravenous chemotherapy only. (HR 0.81 CI 95% 0.74–0.88 *p* < 0.00001) (Figure 2).

**Figure 3:**
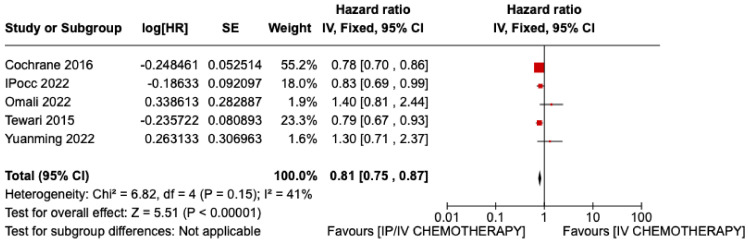
Statistical study of DFS: Results obtained from all studies included except Wright 2015 [13,24,25,26,28].

Similarly, the disease-free interval was significantly greater in the group of patients who underwent intraperitoneal chemotherapy in addition to intravenous chemotherapy (Figure 3) (HR 0.81, CI 95% 0.75–0.87, *p* < 0.00001).

**Figure 4:**
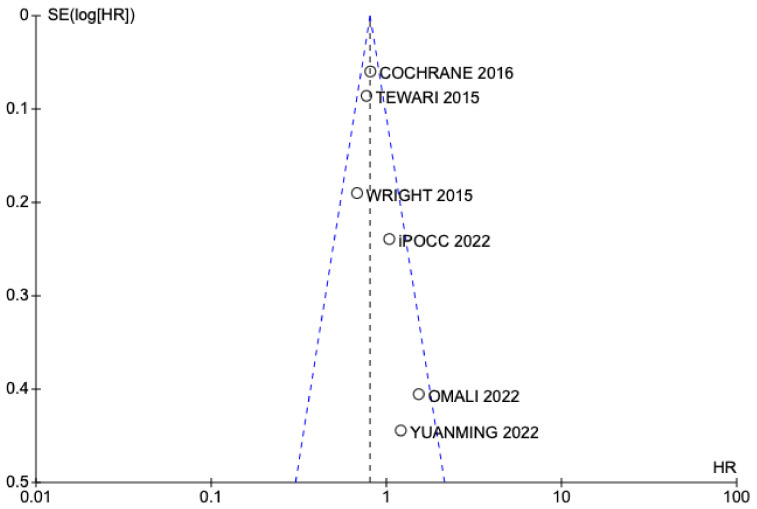
Funnel plot studies included OS [13,24,25,26,27,28].

**Figure 5:**
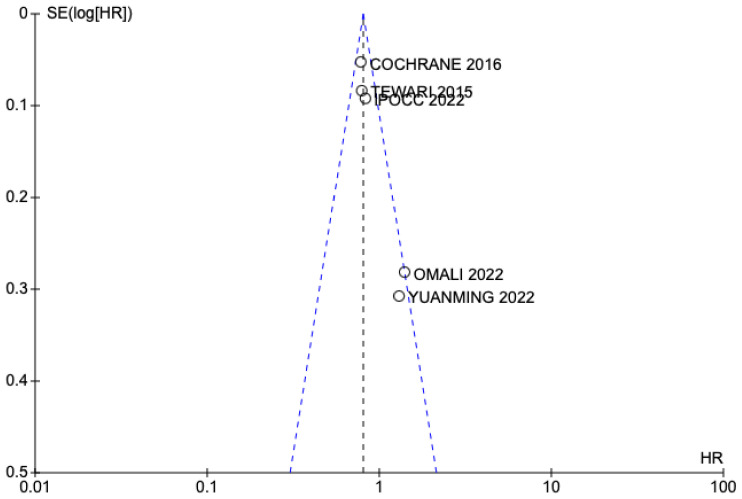
Funnel plot studies included DFS [13,24,25,26,28].

The funnel plot showing the absence of publication bias is shown in Figure 4 and Figure 5. Both graphs show a correct symmetry, which supports the selection of articles according to the established inclusion criteria, regardless of their results, and increases the statistical power of the results obtained.

Although the aims of three articles included assessing the toxicity associated with intraperitoneal treatment, they did not supply values for relative risk (RR).

The results of the iPocc study [24] are not published enterally, but the results we obtained from the abstract published in the SGO Annual Meeting on Womens’ Cancer are, 2022. This abstract specifies similar profiles of toxicity in both groups, but with an increase in catheter-related complications (11.8%).

Yuanming et al. [28] compared the total number of cycles completed in the group with additional IP chemotherapy with those receiving IV chemotherapy. They reported that 93% of intraperitoneal chemotherapy cycles were completed compared with 92% in the intravenous chemotherapy group.

Similarly, Wright et al. [27] reported that the percentage of completed cycles in the intraperitoneal group was 81%, lower than the 91% in the IV chemotherapy group, but with no statistically significant differences in G3/G4 complications.

In contrast, the Cochrane systematic review reported an increased RR of fever, fatigue, infection, gastrointestinal or metabolic disturbances, pain, and hearing loss [25].

## 4. Discussion

Our study shows a statistically significant increase in overall survival and disease-free survival in those patients with advanced ovarian cancer who have been treated with intraperitoneal chemotherapy.

Of the included studies only two, Omali et al. [26] and Yuanming et al. [28], reported no benefit with the use of intraperitoneal chemotherapy. Omali et al. included patients in the GOG 104, 114, and 172 studies, and they report on long-term survivors (>10 years of survival). The use of intraperitoneal chemotherapy was not found to be an independent survival factor. They also included patients who were treated with cyclophosphamide in the intravenous arm, although this that is not first line in the treatment of ovarian cancer [26]. Similarly, Yuanming et al. used a lower dose in the IP group than that used in previous studies, and it is possible that the expected benefits of this treatment were not evident as the theoretical positive effect of this route of administration is based on the cumulative effect and dose in peritoneal cavity assuming an increase in side effects. Moreover, 19 patients included in the IP chemotherapy group were randomized to a single IP dose, which was not demonstrated to be clinically efficacious, although there was a decrease in toxicity.

Tewari et al. [13] only included patients from the studies GOG 114 and GOG 172 because, although GOG 104 reported improved survival in the IP chemotherapy group, cyclophosphamide was administered instead of paclitaxel in the IV group, and it can be assumed that the benefit associated with IP administration in the study may be a consequence of this different treatment regimen in the IV arm.

It should be noted that the population evaluated in this study was not the same as in the study by Omali et al.; Tewari et al. evaluated the entire cohort (847 patients), not only the survivors. They reported an improvement in overall survival, with prognostic impact achieving a tumour residue of less than 1 cm, as well as a 12% reduction in the risk of death with each cycle of chemotherapy administered (HR; 0.88 CI 95% 0.83–0.94).

This difference in the results between these two studies is a consequence of the population analysed; Omali et al. selected long-surviving patients, who would have a theoretically better initial prognosis. It is possible that the beneficial effects of IP chemotherapy are not as evident.

The iPocc trial shows a statistically significant benefit in disease-free survival with the addition of IP chemotherapy but no increase in overall survival [24]. Dense doses of paclitaxel were used without bevacizumab, used in GOG 252, in which there was no evidence of improvement with the use of intraperitoneal chemotherapy, possibly because bevacizumab acts as a confounding factor. In addition, only 42% completed treatment in GOG 172. This is the reason that the dose of cisplatin and paclitaxel was decreased, which could reduce the supposed beneficial effect of intraperitoneal chemotherapy. Similarly, the inclusion of patients with suboptimal surgery and stage IV disease may have increased the positive effect of bevacizumab at the expense of IP chemotherapy, which has been shown to be effective in the subgroup of patients with lower tumour residuals [29].

Of note is the inclusion of patients with tumour residues >2 cm in the iPocc study, in whom this reported benefit was maintained. It is possible that the inclusion of patients with suboptimal surgery results and with stage IV disease does not show the survival benefit of using IP chemotherapy, as previous publications included patients with stage III disease and those with primary surgeries with tumour residues of 1 cm or less.

The use of IP carboplatin is also of interest. Cisplatin has been shown to be a drug with good bioavailability in the peritoneal cavity: peritoneal exposure to the drug reaches 20 times the concentration achieved by intravenous administration. However, carboplatin requires higher doses than cisplatin to achieve these concentrations. Markman et al. noted that the response rate was higher in patients treated with intraperitoneal cisplatin than in those treated with carboplatin [30].

In addition, there was a marked variation in the adoption of IP/IV chemotherapy by articles and significant heterogeneity in the IP/IV regimens used.

Wright et al.’s was the first study to evaluate the clinical applicability of intraperitoneal chemotherapy in clinical studies. However, it used a decreased dose of the drug in the intraperitoneal arm [27].

Retrospective studies have documented higher rates of extra-abdominal cancer recurrences in patients treated with intraperitoneal chemotherapy, raising concerns about whether IP and IV chemotherapy provides effective systemic control [31,32].

Wright et al. reported that women treated with IP/IV chemotherapy had higher distant disease at first recurrence (adjusted rates, 58.8% v 29.4%; AOR, 3.14; 95% CI, 1.34 to 7.36), compared with IV chemotherapy [27]. A possible explanation is the poor systemic control of intraperitoneal chemotherapy. We consider this unlikely due to the current administration of IV treatment at the indicated dose for the adjuvant treatment of AOC. A possible cause is the increase in DFS in the IP arm that may reduce early intra-abdominal recurrences but result in distant recurrences due to the increased time to relapse [27].

The Cochrane review of this topic concluded that intraperitoneal chemotherapy increased OS and DFS in advanced ovarian cancer, but reported worse tolerance, especially in catheter-related complications, such as catheter blockage [25].

GOG 172 is the most widely reported study of catheter-related complications; 36% of patients did not complete treatment due to catheter failure or catheter infection [12].

Three randomised controlled phase III trials (GOG 104, 114, and 172) have demonstrated that IP chemotherapy is an effective treatment for patients with advanced ovarian cancer who have undergone primary optimal surgery (the overall risk of death was reduced by 20–30%).

GOG 172 reported the greatest increase in overall survival in these patients, which increased by 16 months [12,13,17,33,34]. However, intraperitoneal chemotherapy is still rarely used as the first-line treatment in clinical practice due to the high toxicity; inconvenience; risk of catheter-related complications; and lack of a widely accepted optimal regimen [26,27].

Another reason for low rates of use is the lack of standardisation in the dose and timing of this approach which diminishes the available scientific evidence [35].

The main criticism of the GOG studies is the lack of standardisation in the optimal regimen of intraperitoneal treatment. These studies show different intravenous treatment regimens and intraperitoneal treatment regimens, even, in some cases, within arms of the same study, which increases heterogeneity [25,27,28].

GOG 252 showed that reducing the cisplatin dose from 100 to 75 mg/m^2^ in intraperitoneal chemotherapy resulted in a significant decrease in toxicity, but compared with intravenous chemotherapy, intraperitoneal chemotherapy with cisplatin or carboplatin did not result in a survival benefit. A possible explanation for this result is the addition of bevacizumab in both arms [36].

However, in the subgroup analysis comparing patients receiving maintenance bevacizumab with patients not receiving maintenance treatment, there was no evidence of superiority of intraperitoneal chemotherapy over the standard chemotherapy regimen [29].

Although the increased toxicity of intraperitoneal chemotherapy is one of the main reasons for its low rate of use, compliance rates have been shown to be as high as 80% with well-tolerated side effects, even in secondary surgery after relapse [37].

Increased toxicity associated with the use of intraperitoneal chemotherapy was evidenced in the GOG 172 study, where approximately half of the patients did not complete cycles of intraperitoneal chemotherapy due to toxicity [12].

The Cochrane review also reported complications, particularly those related to catheters such as blockage, pain, gastro-intestinal disturbances, and infection [25].

However, the other studies did not report higher rates of side effects in the intraperitoneal treatment arm. In GOG 172, there was no difference in quality of life between treatments groups.

Tewari et al. described age as the main factor associated with compliance with treatment, with a 5% decrease as the patient’s age increased [13].

The NCCN guidelines, which have included IP chemotherapy treatment in patients with advanced FIGO stage III ovarian cancer and optimal cytoreductive surgery as a category 1 recommendation, make special mention of elderly and medicalised patients in whom treatment tolerance is poor [16].

Although the rate of side effects may be greater with intraperitoneal treatment, there was no increase in G3/4 effects. These effects are usually short term and easily treatable.

To minimise toxicity, the included studies varied the IP chemotherapy regimen, but this minimised the statistical power of meta-analysis [12]. For example, 43% of patients in the Wright et al. study received modified regimens [27]. Based on results from Dash et al., a modified dose of day 2 cisplatin (75 vs. 100 mg/m^2^) could reduce toxicity, and 63% of patients received at least five cycles compared with 51% in the GOG 172. However, there are doubts as to whether dose reduction of cisplatin maintains efficacy [38]. To improve tolerance to intraperitoneal treatment, it is necessary to understand the management of complications and to develop an intraperitoneal chemotherapy regimen that combines the described efficacy with acceptable toxicity. On the other hand, in this meta-analysis, we have not evaluated the role of hyperthermia in this subgroup of patients with advanced ovarian cancer.

Hyperthermic intraperitoneal perfusion chemotherapy (HIPEC) is another mode of intraperitoneal chemotherapy delivery that has been proven to be beneficial in the treatment of patients with advanced ovarian cancer treated with neoadjuvant chemotherapy and interval cytoreductive surgery [39].

Hyperthermia has been associated with increased efficacy in intraperitoneal chemotherapy by enhancing its cytotoxic effect and decreasing its toxicity, avoiding complications of long-term peritoneal access [40,41,42].

A systematic review published in 2019 shows that the addition of HIPEC to cytoreductive surgery could significantly improve OS of both primary and recurrent ovarian cancer, although the dosage remains unclear [43].

Another systematic review published in 2023 including 674 patients showed that the use of HIPEC in patients with advanced ovarian cancer treated with neoadjuvant chemotherapy associated with interval surgery has an increase in overall survival and disease-free interval. In addition, the drug administered intraperitoneally with the best results was reported to be cisplatin [44].

A multicentre retrospective cohort study comparing two groups of patients with advanced ovarian cancer undergoing interval surgery alone or in association with HIPEC was published in 2023. After a propensity score, 170 patients in each group were analysed, showing no significant difference in OS but with differences in the DFS [45]. One possible reason for not finding differences in survival is that 53% of patients were treated with paclitaxel, a drug that appears to be less effective than cisplatin, as well as being associated with greater abdominal pain and rates of catheter-related complications.

These results in patients with worse prognosis at baseline who had better survival and disease-free interval outcomes with HIPEC appear to support the potential benefit of first-line IP therapy in advanced ovarian cancer.

A trial studying the role of HIPEC after primary surgery in patients with advanced ovarian cancer is currently in the recruitment phase (OVHIPEC-2) and may provide more information [46].

Another point that has not been evaluated in the present meta-analysis is the role of the BRCA mutation.

BRCA status has not been assessed in this review due to the absence of BRCA status in the included studies. This is one of the main limitations of the study, as previous publications describe a higher sensitivity to intraperitoneal chemotherapy in the subgroup of patients with BRCA mutations [47,48,49].

The use of poly (ADP-ribose) polymerase inhibitors (iPARP) is associated with an increase in DFS, although the magnitude of this effect varies between different patient profiles, showing the best results in the treatment of patients with AOC in the first line and as maintenance treatment in patients with the BRCA mutation or homologous recombination deficiency (HDR) [50].

In the PRIMA and VELIA studies, niraparib is used as a maintenance treatment for patients at a high risk of relapse in the former, excluding patients with complete surgery, and veliparib as the first-line and maintenance treatment in patients regardless of biomarker or type of surgery [51,52].

Treatment with other drugs has also been studied, such as bevacizumab, an anti- vascular endothelial growth factor (anti-VEGF) that is associated with an increase in DFS in patients at high risk of recurrence; FIGO IV; or suboptimal surgery in stage III.

In the PAOLA-1 study, olaparib was added to bevacizumab treatment and was associated with an improvement in DFS regardless of BRCA status [53].

Despite the above results, none of the above therapeutic options have been shown to increase overall survival in patients with advanced ovarian cancer.

Further studies are needed to determine the role of IP chemotherapy in patients; however, it should be noted that since the introduction of IV platinum and taxane-based chemotherapy, prior to GOG 172, no such significant increase has ever been achieved in patients with advanced ovarian cancer.

Limitations:

The main limitation of this study is the heterogeneity resulting from the inclusion of studies whose design did not standardise the chemotherapy treatment used. Different chemotherapy treatment regimens; doses; treatment regimens; and different medications were utilised in the experimental arm.

Another notable limitation is the absence of data on toxicity and side effects. This is the main limitation to the use of intraperitoneal chemotherapy, in addition to the fact that no cost-effectiveness study has been carried out to evaluate the additional costs associated with the use of intraperitoneal chemotherapy.

Strengths:

The main strength of this study is the inclusion of recent studies showing a clear trend of improvement in survival and disease-free period in patients with low tumour residue after optimal cytoreductive surgery, without assuming an increase in toxicity. The side-effects are the main reason that the use of intraperitoneal chemotherapy has not been standardised.

Despite heterogeneity in the treatment regimen and the limitations of our study, we observed a significant survival benefit associated with IP/IV compared with IV chemotherapy. There were few differences in treatment-related toxicities between groups, suggesting that IP/IV chemotherapy is feasible to use in clinical practice.

The optimal treatment of patients with advanced ovarian cancer should be developed by a multidisciplinary team that achieves a good cytoreduction rates, followed by optimal treatment based on platinum and taxane, and to select those patients who benefit from intraperitoneal treatment [7].

Further studies are needed to establish the profile of patients who would benefit from this treatment, to avoid toxicity in patients who would not benefit from it.

Another reason that intraperitoneal chemotherapy has not been a widely used treatment, despite showing an increase in survival comparable to that observed following platinum- and taxane-based chemotherapies, is possibly due to the low amount of promotion of it by pharmaceutical companies, unlike other targeted drugs with a greater economic benefit.

Moreover, the low utilisation of intraperitoneal chemotherapy is due to catheter-related complications such as obstruction or abdominal pain during treatment. These complications and the lack of training of oncologists in their management mean that a large part of the medical oncology community is reluctant to use intraperitoneal chemotherapy in patients with advanced ovarian cancer despite the scientific evidence and the recommendations of clinical guidelines [54].

Improved knowledge of the peritoneum has led to knowledge of the best drug and the optimal intraperitoneal chemotherapy regimen with acceptable toxicity. It should be noted that the benefit observed with intraperitoneal administration has been developed with the use of intravenously administered drugs and it is possible that this improvement in the prognosis of these patients will increase with the development of drugs designed for peritoneal administration considering the physiology of the peritoneum.

In our opinion and based on the results obtained, intraperitoneal chemotherapy should be a treatment to be considered in young patients with little associated morbidity in which complete cytoreduction is achieved or with a tumor residue of less than 1 cm. This patient profile seems to have the most benefit from the treatment and presents the most tolerable toxicity. Likewise, the chemotherapeutic agent of choice would be cisplatin due to its bioavailability at the peritoneal level and the reduced irritation of the peritoneum that it causes.

## 5. Conclusions

In conclusion, this meta-analysis is not based on many articles due to the limited number of publications on this topic in the last 10 years and the heterogenicity in the design of these studies; the results obtained show that IP/IV chemotherapy is a treatment that increases the survival and the time of recurrence. However, it is underused, despite a growing amount of evidence supporting a survival advantage for ovarian cancer patients. There are still issues to be solved, such as the optimal regimen and dosage, the best moment of HIPEC, the optimal patient profile, and the role of biomarkers.

Research is needed on new drugs with a peritoneal route of administration, increasing their effect and decreasing the side effects and their combination with intravenous drugs.

Similarly, it is necessary to determine the possible toxicity associated with the different treatment regimens and establish the best pattern of treatment and the profile of patients who benefit from this treatment.

## Figures and Tables

**Table 1:** Characteristics of included studies.

Included Studies	Type of Trial	Participants	Interventions	Primary Outcome
Cochrane 2016 [25]	Systematic review	OS (2026) DFS (1311)	IP/IV chemotherapy vs. IV chemotherapy	OS DFS Toxicity
IPocc 2022 [24]	Randomised trial of superiority	746	Superiority of IP chemotherapy	DFS
Omali 2022 [26]	Three randomised trials: NRG/GOG 104 NRG/GOG 114 NRG/GOG 172	160 (long-term disease-free survivors (LTDFS)	IP/IV chemotherapy vs. IV chemotherapy	Determine independent prognostic factors of LTDFS.
Wright 2015 [27]	Prospective cohort study	402	IP/IV chemotherapy vs. IV chemotherapy	OS Toxicity
Yuanming 2022 [28]	Retrospective cohort study	255	IP/IV chemotherapy vs. IV chemotherapy	OS DFS Toxicity
Tewari 2015 [13]	Two randomised trials: NRG/GOG 114 NRG/GOG 172	876	IP/IV chemotherapy vs. IV chemotherapy	Long-term survival and associated prognostic factors

**Table 2:** Clinicopathological characteristics of included studies.

Included Studies	Age (Years)	FIGO Stage (III and IV)	Serous Histology	Cytoreductive Surgery (None or <1 cm)	Chemotherapy Regimen	DFS (HR, CI 95%)	OS (HR, CI 95%)
IPocc 2022 [24]	-	87%	64.12%	39.69%	IV: paclitaxel 80 mg/m^2^ + IP: carboplatin AUC 6	0.83 (0.69–0.99)	0.81 (0.75–0.91)
Omali 2022 [26]	57.2	100%	68.4%	33.6%	IV: cyclophosphamide or carboplatin + paclitaxel iv + IP: cisplatin	1.40 (0.81–2.44)	1.53 (0.69–3.38)
Wright 2015 [27]	55–64 (37%)	91%	76%	66%	IV: carboplatin + paclitaxel or docetaxel. IP: cisplatin	N/A	0.68 (0.47–0.99)
Yuanming 2022 [28]	53	100%	90.5%	100%	IV: carboplatin + IP: cisplatin 80 mg ip single dose or 75 mg/m^2^ every 3 weeks	1.30 (0.71–2.37)	1.21 (0.51–2.91)
Cochrane 2016 [25]	N/A					0.78 (0.70–0.86)	0.81 (0.72–0.91)
Tewari 2015 [13]	>55 (485)	100%	72.5%	63.9%	IV: cisplatin + paclitaxel or carboplatin intensive + IP: cisplatin + paclitaxel or paclitaxel	0.79 (0.67–0.93)	0.77 (0.65–0.91)

## Data Availability

Data sharing is not applicable to this article as no new data were created or analysed in this study.

## Supplementary Materials

The following supporting information can be downloaded at: https://www.mdpi.com/article/10.3390/jpm13121636/s1, Figure S1: Prima checklist, Figure S2: Risk of bias summary, Figure S3: Risk of bias graph.
